# Altered oligodendroglia and astroglia in chronic traumatic encephalopathy

**DOI:** 10.1007/s00401-021-02322-2

**Published:** 2021-05-21

**Authors:** K. Blake Chancellor, Sarah E. Chancellor, Joseph E. Duke-Cohan, Bertrand R. Huber, Thor D. Stein, Victor E. Alvarez, Benjamin W. Okaty, Susan M. Dymecki, Ann C. McKee

**Affiliations:** 1 Department of Genetics, Harvard Medical School, 77 Avenue Louis Pasteur, Boston, MA 02115 USA; 2 Alzheimer’s Disease Research and Chronic Traumatic Encephalopathy Center, Boston University School of Medicine, 72 East Concord Street, B-7800, Boston, MA 02118 USA; 3 Department of Neurology, Boston University School of Medicine, 72 East Concord Street, C-3, Boston, MA 02118 USA; 4 Department of Pathology and Laboratory Medicine, VA Boston Healthcare System, 150 S Huntington Ave, #A2-73, Boston, MA 02130 USA; 5 Department of Pathology, Boston University School of Medicine, 670 Albany Street, 4th Floor, Boston, MA 02118 USA; 6 Edith Nourse Rogers VA Medical Center, 200 Springs Road, Bedford, MA 01730 USA

**Keywords:** Chronic traumatic encephalopathy, Single-nucleus transcriptomics, White matter, Neurodegeneration, Oligodendrocytes, Astrocytes

## Abstract

Chronic traumatic encephalopathy (CTE) is a progressive tauopathy found in contact sport athletes, military veterans, and others exposed to repetitive head impacts. White matter rarefaction and axonal loss have been reported in CTE but have not been characterized on a molecular or cellular level. Here, we present RNA sequencing profiles of cell nuclei from postmortem dorsolateral frontal white matter from eight individuals with neuropathologically confirmed CTE and eight age- and sex-matched controls. Analyzing these profiles using unbiased clustering approaches, we identified eighteen transcriptomically distinct cell groups (clusters), reflecting cell types and/or cell states, of which a subset showed differences between CTE and control tissue. Independent in situ methods applied on tissue sections adjacent to that used in the single-nucleus RNA-seq work yielded similar findings. Oligodendrocytes were found to be most severely affected in the CTE white matter samples; they were diminished in number and altered in relative proportions across subtype clusters. Further, the CTE-enriched oligodendrocyte population showed greater abundance of transcripts relevant to iron metabolism and cellular stress response. CTE tissue also demonstrated excessive iron accumulation histologically. In astrocytes, total cell numbers were indistinguishable between CTE and control samples, but transcripts associated with neuroinflammation were elevated in the CTE astrocyte groups compared to controls. These results demonstrate specific molecular and cellular differences in CTE oligodendrocytes and astrocytes and suggest that white matter alterations are a critical aspect of CTE neurodegeneration.

**Supplementary Information:**

The online version contains supplementary material available at 10.1007/s00401-021-02322-2.

## Introduction

Chronic traumatic encephalopathy (CTE) is a progressive tauopathy associated with exposure to repetitive head impacts (RHI) [15, 19, 35, 38–40]. CTE has been reported in contact sport athletes, military veterans and others exposed to neurotrauma [19, 39, 40]. The symptoms of CTE include mood and behavioral abnormalities, cognitive impairment, and dementia [40, 41, 43, 52]; however, like most neurodegenerative diseases, the diagnosis of CTE can only be made on post-mortem neuropathological evaluation, and there are no treatments. A National Institute of Neurological Disease and Stroke (NINDS)/National Institute of Biomedical Imaging and Bioengineering (NIBIB) panel defined the pathognomonic lesion of CTE as the perivascular accumulation of hyperphosphorylated tau (p-tau) in neurons and astrocytes in an irregular pattern in the cerebral cortex, most prominent at the depths of the sulci [38]. These criteria were recently confirmed by a second NINDS–NIBIB consensus panel who further concluded that a single pathognomonic lesion was sufficient for diagnosis and that p-tau pathology in neurons was a necessary component of the pathognomonic lesion [7]. CTE is distinguished from other tauopathies by a distinctive p-tau pathology that primarily involves the frontal and temporal cortices, medial temporal lobe, and brainstem although there are also profound and widespread changes in the subcortical white matter [39, 40]. White matter abnormalities occur early in CTE and include white matter rarefaction, myelin and axonal loss, astrocytic degeneration, microglial inflammation, microvasculopathy, and perivascular hemosiderin-laden macrophage deposition [16, 19, 23, 25, 40, 53]. In addition, white matter rarefaction is independently associated with dementia in CTE suggesting that white matter alterations might play a key role in the production of clinical symptoms [2, 13, 40]. To date, scientific advances in CTE have focused on CTE neuropathology, postmortem diagnostic criteria, clinicopathological correlation, proposed diagnostic criteria for traumatic encephalopathy syndrome (TES), and biomarkers to detect CTE during life; few studies have explored alterations at the molecular and cellular levels.

Previous transcriptomic analyses of CTE have identified dysregulated neuronal genes, including the protein phosphatase-encoding gene *PPP3CA* [48], as well as alterations in genes encoding synaptotagmin 1, calcium/calmodulin-dependent protein kinase II, protein kinase A, protein kinase C, and cell adhesion molecules[14]. Quantitative proteomic analysis of dorsolateral frontal (DLF) gray and white matter reported reduced levels of axonal signaling pathway proteins and Western blot analysis showed significant reduction in protein levels of oligodendrocyte- (OL-) specific proteins (such as myelin associated glycoprotein, myelin basic protein, and tubulin βIV) and neuronal markers such as cofilin-1 and tubulin βIII [6]. These findings, together with neuropathological observations of microvascular pathology and inflammation in the white matter, implicate glial alterations as factors contributing to the neurodegenerative process in CTE. To date, however, no studies have systematically analyzed the differential vulnerabilities of individual glial cell types in CTE using single-nucleus RNA-sequencing (snRNA-seq) [21].

To isolate glial cells for analysis, we explored cell-type-specific mRNA profiles in white matter from subjects with neuropathologically-verified CTE compared to age-, sex, and post-mortem interval (PMI)-matched controls by applying the method of snRNA-seq [20, 27, 36, 47, 55], in combination with complementary in situ histology, immunohistochemistry (IHC), immunofluorescence (IF), and single-molecule fluorescent mRNA in situ hybridization (smFISH) validation techniques. Our transcriptomic analyses yielded 24,735 individual brain nuclei RNA profiles. Similar to previous reports using snRNA-seq in other neurological diseases and disorders, including Alzheimer’s disease (AD), multiple sclerosis (MS), autism spectrum disorder (ASD), Huntington disease (HD), and epilepsy [1, 20, 27, 36, 47, 55], our findings, the first at single nucleus resolution, indicate differences in cell types, subpopulations, and gene expression in CTE white matter compared to controls. These analyses provide further insight into the white matter alterations that accompany CTE neurodegeneration.

## Materials and methods

### Human brain tissue

#### Controls

Selection of control cases from the VA National Posttraumatic Stress Disorder (PTSD) brain bank at VA Boston Healthcare System was based on absence of a clinical diagnosis of PTSD or any other neurological or mental health disorder and availability of fresh frozen tissue from dorsolateral frontal (DLF) cortex (Brodmann area 8/9). Control cases were also neuropathologically free of neurological or neurodegenerative disease and had no history of traumatic brain injury (TBI), physical abuse or other trauma. There was no available information regarding contact sports participation or exposure to RHI.

#### CTE

Brain tissue samples of fresh frozen dorsolateral frontal (DLF) cortex (Brodmann area 8/9) were selected from the Understanding Neurological Injury and Traumatic Encephalopathy (UNITE)/ Veterans Affairs-Boston University-Concussion Legacy Foundation (VA-BU-CLF) brain bank at VA Boston Healthcare System. Selection of cases was based on matching age, gender, PMI and RIN to controls, availability of fresh frozen DLF tissue, neuropathological diagnosis of stage II or III CTE, and the absence of co-morbidities, including: no infarcts, lacunes or microinfarcts, no Lewy bodies or alpha-synuclein immunopositivity, none-sparse diffuse beta amyloid plaques, no neuritic plaques, none-low Alzheimer’s disease neuropathological change (ADNC), no hippocampal sclerosis and none-sparse TDP-43. Selection of cases was performed blinded to all database information regarding white matter integrity, including white matter rarefaction, cribriform state and density of perivascular hemosiderin-laden macrophages (Table 1). The UNITE and PTSD brain banks use the same laboratory and research staff to perform the neuropathological processing, analysis and storage of brain tissue which ensures that the tissue metrics for each bank are identical. CTE and control brain tissue samples were matched by age (Control: 55.6 ± 7.5 years; CTE: 55.9 ± 10.7 years; *P* = 0.94, two-tailed Mann–Whitney *U* test), PMI (Control: 31.7 ± 9.7 h; CTE: 35.3 ± 10.2 h; *P* = 0.70, two-tailed Mann–Whitney *U* test), and RIN values (Control: 6.4 ± 1.7; CTE: 6.8 ± 1.7; *P* = 0.56, two-tailed Mann Whitney *U* test) (Appendix Fig. 5b, c, d). Clinical data and demographics of the CTE and control brain tissue donors are provided in Table 1.

**Table 1:** Demographic and clinical profiles of subjects

Subject	Age	Sport exposure	Years of exposure	TBI	CTE stage	WMR	Cribriform state	WM perivascular macrophages	COD	PMI (h)
CTE 1	65	NFL	17	0	III	0	0	1	Cardiovascular	37
CTE 2	69	NFL	15	0	III	3	0	2	GI cancer	24
CTE 3	52	NFL	21	0	III	2	3	3	Liver failure	48
CTE 4	70	NFL	15	0	II	1	0	1	Cardiovascular	31
CTE 5	38	NFL	20	0	II	0	0	2	Cardiovascular	24
CTE 6	49	MMA/BOX	16	0	II	0	0	3	GI cancer	24
CTE 7	47	COLFB	12	0	III	1	0	1	Pancreatitis	47
CTE 8	57	USFL	11	0	III	2	1	2	Liver failure	47
CONTROL 1	65	NR	NR	0	NA	0	0	0	Cardiovascular	27
CONTROL 2	60	NR	NR	0	NA	0	0	0	Thromboembolism	30
CONTROL 3	53	NR	NR	0	NA	0	0	0	Accident	36
CONTROL 4	69	NR	NR	0	NA	0	0	0	Suicide	38
CONTROL 5	48	NR	NR	0	NA	0	0	0	Accident	11
CONTROL 6	51	NR	NR	0	NA	0	0	0	Cardiovascular	29
CONTROL 7	48	NR	NR	0	NA	0	0	0	Cardiovascular	45
CONTROL 8	51	NR	NR	0	NA	0	0	0	Accident	39

*NFL* National Football League, *MMA* mixed martial arts, B*O*X boxing, *COLFB* college football, *USFL* United States Football League, *NR* not recorded, *TBI* traumatic brain injury, *WMR* white matter rarefaction, cribriform state, *WM* white matter, *COD* cause of death, *PMI* post-mortem interval

#### Single-nucleus RNA-seq analysis

The subcortical white matter was dissected from the tissue blocks and included both deep white matter and U-fibers. For the in situ analysis, DLF tissue with both cortical and white matter tissue adjacent to the white matter block taken for snRNA-seq analysis was isolated (Appendix Fig. 5a). RIN number determination was completed according to manufacturer instructions using a Bioanalyzer RNA 6000 Nano kit (Agilent).

### Isolation of cell nuclei

All tissue was dissected into 30 mg sections from DLF white matter with a scalpel blade and stored at − 80° C. On the day of single-nucleus encapsulation, tissue was placed in a 7 mL dounce with ice cold buffer (0.5 M sucrose, 2 M KCl, 1 M MgCl2, 1 M Tricine-KOH pH 7.8, spermine, spermidine, DTT, RNasin, H2O) and dounced with a tight pestle ten times to mechanically dissociate and homogenize the tissue. To further dissociate the tissue, 5% IGEPAL-CA630 was added to the homogenate and dounced 5 times. The homogenate was then strained through a 40 μm cell strainer (VWR) on ice. 5 mL of 50% iodixanol was added to the homogenate and mixed. In a 13 mL ultraclear ultracentrifuge tube, 1 mL of 40% iodixanol was added with 1 mL of 30% iodixanol carefully layered on top, followed by 10 mL of the tissue homogenate. The tissue was then ultracentrifuged in a swing-bucket rotor at 10,000xg for 18 min. Following ultracentrifugation, the 35% iodixanol layer was collected and the nuclei were counted. Nuclei were diluted with 30% iodixanol to 90,000 nuclei/mL for inDrops chip loading. Nuclei were isolated for inDrops using a previously described method [24].

### Droplet-based snRNA-seq with inDrops and sequencing

Isolated nuclei were delivered to the inDrops single cell core facility at Harvard Medical School. The inDrops core facility generated inDrops V3 nuclei libraries for each subject by encapsulating each nucleus in a hydrogel containing barcodes and unique molecular identifiers (UMIs). Nuclei were lysed and each free mRNA molecule was reverse transcribed to cDNA and labeled with a barcode and UMI, which enables mapping that specific cDNA molecule to its particular cell of origin and ensures that specific amplified sequence is counted only once bioinformatically [29, 58], thus avoiding the production of potential PCR amplification-generated artifact. This process produced labeled cDNA molecules for all 16 samples. Sample cDNA was delivered to the Biopolymers Facility at Harvard Medical School. Sample quality was assessed by Agilent Tapestation. Samples with excess primer dimer contamination were filtered by SPRIselect size selection (Beckman Coulter, B23317), followed by pooling of libraries and dilution for sequencing. Pooled CTE and control samples were sequenced on the Illumina NextSeq500 at an average of 40,000 reads per nucleus barcode, approximately 360,000,000 reads per sample.

### Pre-processing snRNA-seq sequencing data

The Biopolymers Core Facility delivered Binary Base Call (BCL) files for each sequencing run, in which each run contained an equal number of matched CTE and control samples. The inDrops single cell method requires four primers to completely capture and de-multiplex a cDNA molecule. The first primer captures the transcript, the second captures part of the single cell barcode, the third captures the library or sample index, and the fourth captures the rest of the single cell barcode, the UMI, and the polyT tail. BCL files produced by sequencing were converted to 4 FastQ files, one per inDrops primer read. FastQ files were de-multiplexed by first filtering reads, only keeping reads that followed the expected inDrops index structure, described above. Reads that passed structure filtering were de-multiplexed based on library index, resulting in reads by sample of origin, i.e., subject. Abundant barcodes were identified and sorted so that each cDNA from a single nucleus was grouped together. After each read was de-multiplexed, thus assigning reads to nucleus of origin and nuclei to sample of origin, reads were aligned with bowtie/1.1.1 RRID:SCR_005476 to an edited version of the GRCh38.97 reference genome so that all exonic and intronic reads were counted. Aligned reads were quantified and a UMI count matrix was produced for each sample. De-multiplexing was executed following the inDrops analysis pipeline (https://github.com/indrops/indrops).

### Quality control and cell filtering

UMI count matrices for each subject were loaded into R version 3.6.1 RRID:SCR_001905 using the Scater R package RRID:SCR_015954 [37]. Control samples were combined and CTE samples were combined to generate two separate objects with the R package Seurat version 3.1 RRID:SCR_007322 [9]. The Seurat object filtration process included excluding nuclei with fewer than 400 genes and more than 2,500 genes, which limited our analysis to nuclei with high-quality RNA. We also removed nuclei with more than 1% mitochondrial genes, which excluded nuclei that were potentially undergoing lysis due to sample preparation. To identify cell types and/or cell states in the data set, we constructed an unbiased *k*-nearest neighbor graph based on top principal components followed by a Jaccard similarity step and the Louvain modularity optimization algorithm [8] to group the cell nuclei into clusters based on gene expression similarities and produce a two-dimensional t-distributed stochastic neighbor embedding (tSNE) projection [9, 34]. The tSNE algorithm provides an unbiased similarity clustering of nuclei by gene expression, shaped by parameters selected prior to analysis, such as number of principal components, number of highly variable genes, and resolution [30]. Through iterative parameter space testing [20, 27, 30, 36, 47, 55], we settled on values that resulted in stable clustering with high intercluster distance and low intracluster distance, meaning, on average, nuclei in the same cluster were more similar to each other than to nuclei in a different cluster. Specifically, we chose 23 principal components and a resolution of 0.8 for input parameters (Appendix Fig. 5h). The Seurat objects were then normalized and log-transformed using Seurat’s implementation of SCTransform and integrated and corrected for batch effects using Seurat’s implementation of canonical correlation analysis [9, 22] (CCA). Integration was performed to integrate control and CTE samples, thus creating a single Seurat object for downstream analyses, “CTE.combined.”

### Cell clustering

The single integrated Seurat object, “CTE.combined,” was scaled using ScaleData and a principal component analysis (PCA) was performed for initial dimensionality reduction. An Elbow Plot was produced to determine the number of principal components to be used for further analyses (Appendix Fig. 5h). Based on the Elbow Plot, 23 principal components were used. Using RunTSNE, FindNeighbors, and FindClusters, “CTE.combined” was further reduced in dimensionality to produce a two-dimensional t-distributed stochastic neighbor embedding (tSNE) projection. This process included constructing an unbiased *k*-nearest neighbor graph based on top principal components followed by a Jaccard similarity step and the Louvain modularity optimization algorithm. Parameters, including resolution, were varied over several iterations to identify a tSNE that was in agreement with published human white matter brain data [27] and that represented nuclei clusters that were consistently present over several iterations. Nuclei distances were also considered to increase intercluster distances and decrease intracluster distances, thereby producing clusters in which nuclei in the same cluster were more similar to each other than to nuclei in a different cluster. The final tSNE projection used was produced at resolution 0.8 and included 18 transcriptionally distinct nuclei clusters.

### Cell-type annotation and sub-clustering

**Fig. 1:**
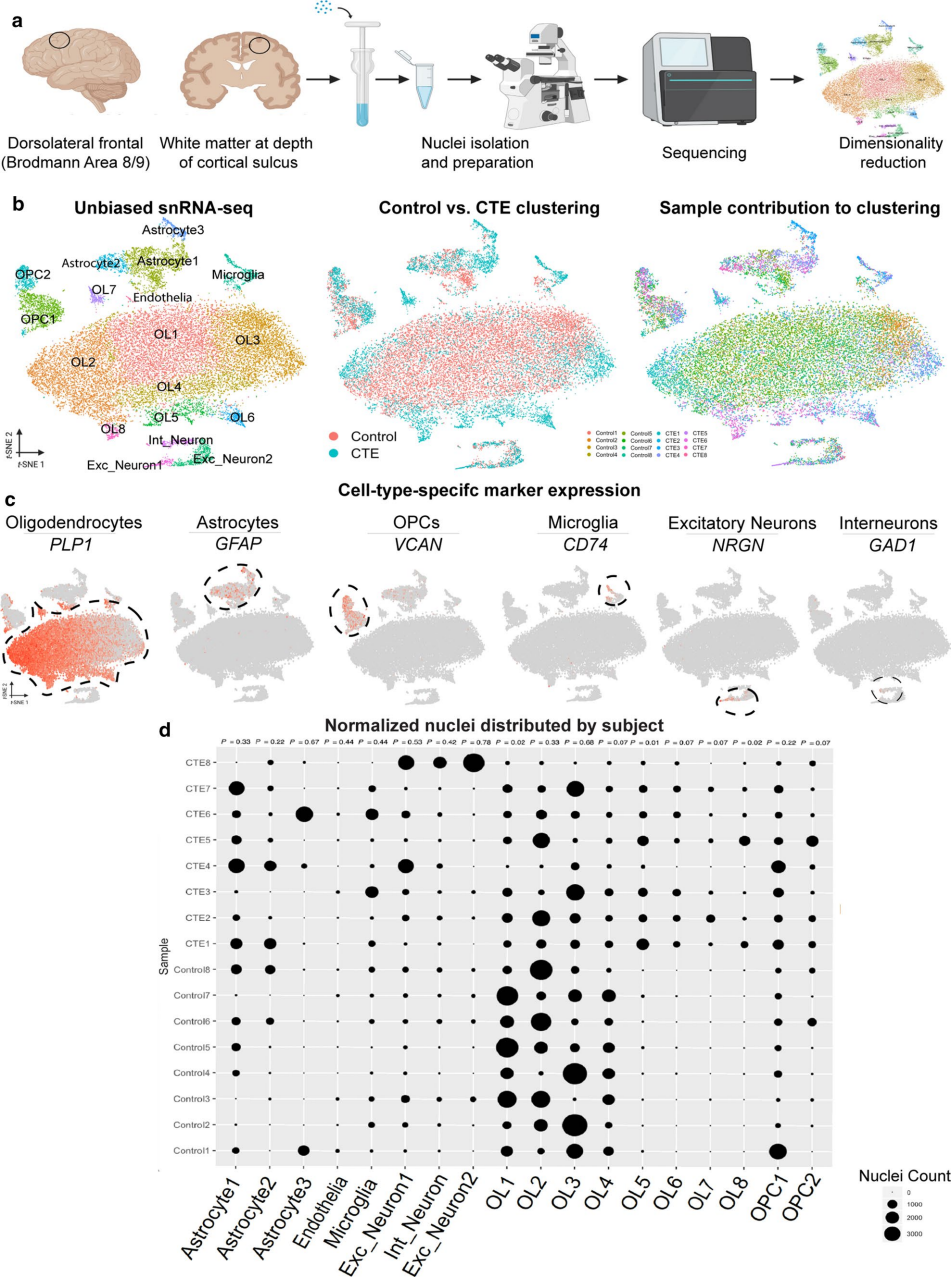
Overview of snRNA-seq and experimental approach. **a** Dorsolateral frontal cortex area. White matter taken for snRNA-seq analysis and validation. Dounce homogenizer for tissue disassociation. Inverted microscope for single-nucleus hydrogel encapsulation. Sequencer and example data. **b** (Left) Unbiased tSNE showing all major cell-types expected in human brain tissue. (Middle) tSNE colored by condition, red: Control, teal: CTE. (Right) tSNE colored by subject, figure key below. **c** tSNE projection of each major cell type colored by expression of canonical marker genes. Nuclei clusters positive for each marker gene are circled by a dashed line.** d** Bubble plot of normalized number of nuclei with each subject in the data set on the *y*-axis and each cell-type and subpopulation on the *x*-axis. Size of the bubble indicates the number of normalized nuclei in each cell-type for each subject. *P* values for all nuclei subpopulations listed at top (*n* = 8 per condition; Astrocyte1: *U* = 19.5; Astrocyte2: *U* = 16.5; Astrocyte3: *U* = 27.0; Endothelia: *U* = 22.5; Microglia: *U* = 22.0; Exc_Neuron1: *U* = 24.0; Int_Neuron: *U* = 21.0; Exc_Neuron2: *U* = 29.0; OL1: *U* = 6.0; OL2: *U* = 19.0; OL3: *U* = 27.0; OL4: *U* = 10.0; OL5: *U* = 0.0; OL6: *U* = 5.5; OL7: *U* = 11.0; OL8: *U* = 5.5; OPC1: *P* = 0.22, *U* = 16.0; OPC2: *P* = 0.07, *U* = 11.0; FDR-corrected two-tailed Mann–Whitney *U* test)

Nuclei clusters were annotated using canonical cell-type marker genes: *PLP1, MBP,* and *CLDN11* for OLs; *GFAP* and *AQP4* for astrocytes; *VCAN* and *PDGFR*α for oligodendrocyte precursor cells (OPCs); *CD74* and *ITGAM* for microglia, *NRGN* and *SLC17A7* for excitatory neurons; *GAD1* and *GAD2* for interneurons; and *CLDN5* for endothelial cells. FindAllMarkers and FindMarkers were used to identify cluster-specific genes [20, 27, 36, 47]. Top genes for each cluster and the expression of top genes on the tSNE were used to identify clusters (Fig. 1c, Appendix Fig. 6, SI 2). To further analyze specific cell-types, clusters from the primary tSNE were subset. SubsetData was used to isolate nuclei from a specific cell-type, and ScaleData, RunPCA, RunTSNE, FindNeighbors, and FindClusters were performed on the subset data to produce a new Seurat object for each cell-type.

### Cell-type-specific gene expression analysis and differential gene expression analysis

The Seurat function FindMarkers was used to determine the marker genes (the genes that make a specific cluster unique compared to the other clusters) in any given cluster or subset cluster (SI 2). Differential gene expression analysis was performed using Seurat’s implementation of MAST (SI 3) [18].

### Technique correlation analysis

To determine whether findings across experimental methods were significantly correlated, normalized nuclei counts identified by snRNA-seq transcriptomic analysis were plotted against the number of *OLIG2* + nuclei identified by smFISH. Each dot on the plot represented a single human subject (Control: Red, CTE: Teal), with snRNA-seq transcriptomic analysis providing the x-coordinate value and smFISH providing the y-coordinate value. A Shapiro–Wilk test was performed to determine normality of the data set, and a Pearson correlation coefficient was produced to determine correlation and statistical significance.

### Gene ontology analysis

The set of all genes significantly differentially expressed between CTE and control nuclei (FDR < 0.05) within OL and astrocyte cell-types was used as query inputs for gene ontology analyses by PANTHER Classification System v14.1 (www.pantherdb.org) RRID:SCR_004869 [42] to identify over-represented gene modules. Modules were assigned by PANTHER's statistical overrepresentation test in which a binomial test was applied to each query set relative to its corresponding reference gene set (all genes expressed in a certain cell-type) to determine if a known biological pathway had non-random linkage within the query set. All identified modules were derived from PANTHER's "GO biological process complete,” “GO cellular component complete,” “GO molecular process complete,” and “Reactome Pathways” gene annotation and only those with a false-discovery-rate-corrected *P*-value < 0.05 were considered. Gene modules with the highest fold enrichment values were considered.

### Cell-type representation analysis

To determine whether any cluster of nuclei was increased or decreased in CTE samples, compared to control subjects, numbers of nuclei in each cluster were normalized to the sample with the largest number of nuclei (see methods in [47], SI 5). The following formulas for normalization were used:

$${\text{Normalization factor}} = \frac{{\text{Total nuclei in sample}}}{{\text{Total nuclei in sample with the largest number of nuclei}}},$$

$${\text{Normalized cell number}} = \frac{{\text{Raw cell number in a cluster}}}{{\text{Normalization factor}}}.$$

Normalized cell numbers for each cell-type and sample were then compared between conditions using a two-tailed Mann–Whitney *U* test (FDR-corrected for multiple comparisons).

### Immunofluorescence

Fresh frozen tissue stored at − 80° C was adhered to cryostat compatible metal chucks using Optimal Cutting Temperature compound (Leica) and allowed to acclimate to a − 17° C cryostat (Leica CM3050 S) for 1 h before cutting. Slides used for IF staining were cut at 10 µm using a CryoJane Tape Transfer System (Leica). Slides were placed in ice cold formalin for 15 min after cutting, rinsed in PBS, and allowed to dry fully under a ventilation hood. Slides were then stored at − 80° C and allowed to thaw at 4° C before being used for manual IF staining. Anti-mouse GFAP (1:1,000; Millipore) Cat# IF03L RRID:AB_2294571 and anti-rabbit CD44 (1:2,000; Abcam) Cat# 1998-1, RRID:AB_764499 antibodies were used for double-labeled IF staining. One slide from every case was placed in three changes of 0.01% Triton for 5 min for tissue permeabilization, blocked in 3% normal donkey serum for 30 min, heated at 95° C in AR6 solution (PerkinElmer) for 20 min, and placed in a 4° C refrigerator overnight with primary antibody. A donkey anti-mouse or a donkey anti-rabbit secondary was used at a 1:500 concentration with 1% Normal Donkey Serum for one hour at room temperature. Fluorescent labeling was conducted using Opal 480 (GFAP) and Opal 570 (CD44) dyes (1:150; Akoya Biosciences) for 10 min at room temperature, followed by 5 min of DAPI counterstaining.

### Immunohistochemistry

Fresh frozen tissue stored at −80° C was adhered to cryostat compatible metal chucks using Optimal Cutting Temperature (OCT) compound (Leica) and allowed to acclimate to a − 17° C cryostat (Leica CM3050 S) for 1 h before cutting. Tissue was cut at 16 µm thick sections and placed on Fisher Superfrost Plus (Fisher Scientific) slides. One slide for every case was heated in a 60° C oven for 15 min after cutting for tissue adherence and placed directly in room temperature 10% formalin for 1.5 h for fixation. Slides were then dehydrated in 50%, 70%, and 100% ethanol baths for 2 min each before being heated in a 60° C oven for 30 min to dry slides for room temperature storage. All brightfield immunohistochemistry was completed using individually optimized protocols using Research Detection Kits on a BOND RX (Leica) system. Antibodies used on the BOND RX included: anti-rabbit PDGFRα (1:100; Abcam) Cat# ab71009, RRID:AB_2162475 and anti-rabbit OLIG2 (1:100; Millipore) Cat# AB15328, RRID:AB_2299035.

### Iron staining

Tissue processing and cutting was conducted in an identical protocol to that of immunohistochemistry. Prussian blue staining, which produces a dark blue pigment on all ferrous iron present in the tissue, was completed on all slides in the same run with hematoxylin and eosin counterstain. Prussian blue stained slides were placed in a solution of 0.06 N Hydrochloric acid and Potassium Ferricyanide for 1 h at room temperature before a wash in 1% Acetic Acid and were counterstained with hematoxylin and eosin.

### Single-molecule fluorescent mRNA in situ hybridization

Tissue processing and cutting were conducted in an identical protocol to that of immunohistochemistry. Single-molecule fluorescent mRNA in situ hybridization was conducted using RNAscope (Advanced Cell Diagnostics) probes and kits optimized for use on a BOND RX (Leica) system. One slide from every case was treated with a 30 min protease step, 1 min of hybridization, and 10 min of epitope retrieval at the beginning and end of the protocol. Concentration of Opal dyes (480, 570, 690; Akoya Biosciences) for fluorescent labeling were diluted at 1:1,500 in TSA Buffer (Advanced Cell Diagnostics) for every probe, all other steps were completed according to manufacturer's instructions.

### Slide visualization and quantification

All slides used for iron staining, IF, IHC, smFISH, and iron staining were scanned at 40× using custom protocols on a Vectra Polaris (Akoya Biosciences) whole slide scanner and quantified using custom protocols on HALO (Indica Labs) software. For IHC analysis, HALO was manually trained to identify the color of the antibody-positive chromogen or dye (DAB, fast red, or Prussian Blue) and counterstain (hematoxylin). By manually adjusting HALO settings on stain color identification and expected inclusion shape and size, chromogen- or dye-positive inclusion counts and/or positively staining area measurements were generated for the selected tissue analysis area. Masks representing the HALO analysis were produced to determine the accuracy of all analyses. For IF and smFISH, HALO is programmed innately to detect the Opal dyes and DAPI used in these experiments. For IF analysis, HALO settings on Opal dye intensity were adjusted to accurately map observed GFAP and CD44 expression. HALO produced values representing the area of GFAP or CD44 positivity based on the manually adjusted positivity settings in the selected tissue analysis area. For RNAscope analysis, HALO settings on dye intensity and inclusion shape were adjusted to identify and map DAPI positive nuclei. HALO settings on Opal dye intensity were adjusted to accurately encompass Opal dye-positive inclusions inside of nuclei and/or within a 5 µm radius from the center of nuclei. This value was selected to best represent measured cell bodies observed in our OLIG2 IHC and GFAP IF staining. Inclusions detected within the set parameters were measured by area. Masks representing the HALO analysis were produced to determine the accuracy of all analyses. All analyses were conducted in 1 mm^2^ sections of the white matter tissue adjacent to the depth of a cortical sulcus unless otherwise specified. Full white matter analyses were conducted on OLIG2 and PDGFRα IHC staining.

### Statistical analyses

Statistical analyses were performed in either R version 3.6.1 RRID:SCR_001905 or GraphPad Prism 8 RRID:SCR_002798. *P* values were considered significant if *α* ≤ 0.05. Gene expression statistical analyses were performed in R using MAST and Bonferroni correction for multiple comparisons [18]. To identify an increase or decrease in the number of normalized nuclei between conditions, a Mann–Whitney *U* test was performed and *P* values were FDR-corrected for multiple comparisons as previously described [47]. IHC, IF, and smFISH quantification was performed using a one-tailed Student’s *t* test or one-tailed Mann–Whitney *U* test, as determined by Shapiro–Wilk normality testing of each analysis. One-tailed tests were performed on all imaging analyses to determine the validity of snRNA-seq findings of significant gene expression and/or nuclei number differences between CTE and control. No statistical methods were used to predetermine sample size. Sample size was determined by tissue availability.

### Figures

All figures were created in Adobe Illustrator version 24.1.2 RRID:SCR_010279.

### Code availability

Source code used for all computational analyses can be found on GitHub at: https://github.com/blakechancellor/Altered-oligodendroglia-and-astroglia-in-chronic-traumatic-encephalopathy.

### Data availability

The snRNA-seq and in situ validation data that support the findings of this study are available from the corresponding author. Raw and processed snRNA-seq data are available at Gene Expression Omnibus (GEO) RRID:SCR_005012 under accession number GSE155114.

## Results

### Single-nucleus RNA sequencing of human postmortem CTE white matter

We used the inDrops droplet-based microfluidics method to encapsulate each nucleus for sequencing [29]. Following sequencing, we performed quality control, cell filtering, and computational analysis using the Seurat pipeline [9, 22] to produce single-nucleus transcriptomic profiles of the 8 control and 8 CTE samples (Fig. 1a, b, SI 1, Methods). We identified a total of 24,735 high-quality, droplet-based snRNA-seq profiles across the 16 brain tissue samples with a median of 561 genes and 854 unique molecular identifiers (UMIs) per nucleus (Fig. 1, Appendix Fig. 5e–g, SI 1). Principal component dimensionality reduction and integrated t-distributed stochastic neighbor embedding (tSNE) of control and CTE single-nucleus transcriptomic profiles yielded 18 transcriptomically distinct nuclei clusters (Fig. 1b, left). Nuclei clusters were evaluated for the presence of gene transcripts encoding canonical cell-identity markers (e.g., *PLP1, MBP,* and *CLDN11* for OLs; *GFAP* and *AQP4* for astrocytes; *VCAN* and *PDGFR*α for OPCs; *CD74* and *ITGAM* for microglia, *NRGN* and *SLC17A7* for excitatory neurons; *GAD1* and *GAD2* for interneurons; and *CLDN5* for endothelial cells). Clusters with a significant increase in expression of transcripts encoding canonical cell-identity markers were labeled with the corresponding cell type (Fig. 1c, Appendix Fig. 6, SI 2, Methods). When control and CTE brain tissue samples were combined, a total of 18,491 OLs (~ 75%), 2,598 astrocytes (~ 11%), 1,879 OPCs (~ 8%), 596 microglia (~ 2%), 857 excitatory neurons (~ 3%), 271 interneurons (~ 1%), and 43 endothelial cells (~ 0.2%) were identified.

Examination of the tSNE projection anchored with cell-identity markers suggested multiple differences in CTE as compared to control samples when split by condition (Fig. 1b, middle). CTE brain tissue samples contained fewer OLs, altered ratios of OL subpopulations, and reduced astrocyte subpopulation heterogeneity. To determine whether these observed differences between CTE and control tissue partially reflected subject-specific variation rather than condition, we normalized nuclei numbers to the subject with the highest number of nuclei (see [47], Methods) and compared the distributions of total nuclei in each cluster by subject (Fig. 1d). While we did observe subject-specific variation in the proportion of nuclei comprising each cluster, when nuclei numbers were normalized, statistically significant differences between CTE and control samples remained. Specifically, we observed the presence of OL subpopulations that were significantly elevated in CTE [17, 27].

### Molecular profiling points to fewer OLs in CTE white matter compared to control

**Fig. 2:**
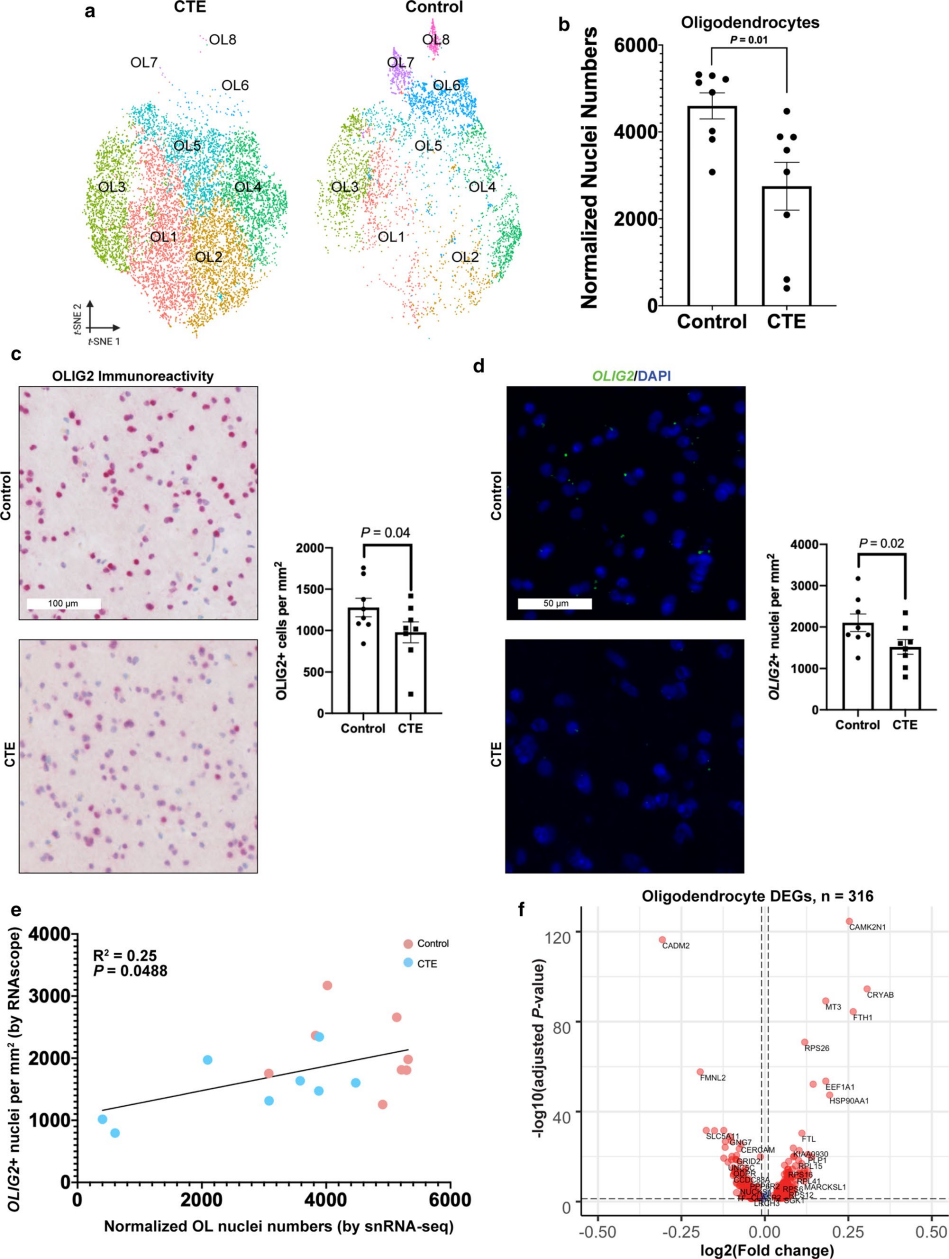
Fewer OLs in CTE white matter compared to control **a** tSNE projection of oligodendrocyte nuclei subset from primary tSNE (Fig. 1b). **b** Scatter plot with bar for normalized nuclei counts for all oligodendrocyte lineage nuclei, dot represents the total normalized number of oligodendrocyte lineage nuclei in one subject (*n* = 8 per condition, median of CTE = 3331, median of control = 5023, *P* = 0.01, FDR-corrected two-tailed Mann–Whitney *U* test, *U* = 9.0). **c** Representative immunohistochemistry images (anti-OLIG2 for oligodendrocyte lineage cells) of control and CTE tissue. Scale bar (top, white): 100 µm. Quantification (left) for number of OLIG2-positive nuclei per mm^2^ over the entire white matter sample for each subject (*n* = 8 per condition, *P* = 0.0484, one-tailed Student’s *t* test, *t*(14) = 1.780). **d** Representative smFISH images for *OLIG2* (green) and DAPI (blue). Scale bar (top, white): 50 µm. Quantification (left) for number of *OLIG2*-positive nuclei for each subject (*n* = 8 per condition, *P* = 0.03, one-tailed Student’s t test, *t*(14) = 2.103). **e** Scatter plot for number of OLs identified by smFISH and snRNA-seq. Each dot represents one subject. Control: red, CTE: teal. (*r* = 0.4995, *P* = 0.0488, *R*^2^ = 0.25). **f** Volcano plot for differentially expressed OL genes between conditions (Bonferroni correction for multiple comparisons). Vertical dashed lines indicate log2(Fold change) cut-off of 0.01. Horizontal dashed lines indicate −log10(adjusted *P* value) cut off of 0.05. Red dots are CTE-associated differentially expressed OL lineage genes (*n* = 316). All data were presented in Mean ± SEM

To investigate cell-type-specific differences in OLs in CTE compared to controls, we isolated OL nuclei clusters from our primary tSNE projection and re-projected OL nuclei as a subset tSNE by identifying each nucleus previously identified as an OL and performing unbiased graph-based clustering to produce an OL-specific tSNE projection (Fig. 2a) [20, 30]. Consistent with our primary tSNE projection, we observed a statistically significant decrease in the normalized number of OLs in CTE subjects compared to control subjects (*P* = 0.01, two-tailed non-parametric Mann–Whitney *U* test) and the emergence of CTE-specific OL subpopulations (Fig. 2b, SI 5, Methods).

We performed IHC and smFISH to compare cellular and molecular expression in OLs in CTE versus control (Fig. 2c, d, Appendix Fig. 7). Using IHC and smFISH, we also found fewer oligodendrocyte transcription factor 2 protein (OLIG2) positive cells in CTE compared to control, consistent with the transcriptomic findings (IHC: *P* = 0.04, one-tailed parametric Student’s *t* test) (Fig. 2c, Appendix Fig. 7a, b) (smFISH: *P* = 0.02, one-tailed parametric Student’s t test) (Fig. 2d, Appendix Fig. 7c, d). In addition, IHC showed fewer OLs throughout the entire extent of DLF white matter in CTE, a loss that was not restricted to the subcortical U-fiber area (Fig. 2c, Appendix Fig. 7a,b).

Notably, we observed a significant, positive correlation between the number of OLs identified by snRNA-seq and smFISH when tracked by subject (Pearson’s *r* = 0.4995, *P* = 0.0488) (Fig. 2e), suggesting that the normalized nuclei counts accurately reflect in situ cell type abundance. The smFISH *OLIG2* probes also identify OPCs, potentially explaining some of the correlative variance between snRNA-seq and smFISH nuclei numbers (Pearson’s *r* = 0.4995, *P* = 0.0488)) (Fig. 2e). By snRNA-seq, we did not observe differences in OPC numbers in our transcriptomic analysis (*P* = 0.13, two-tailed non-parametric Mann–Whitney *U* test) (Appendix Fig. 8a, b). Furthermore, we performed IHC with anti-PDGFRα, an OPC marker, and found no detectable differences (*P* = 0.23, one-tailed parametric Student’s t test) (Appendix Fig. 8c).

Analysis of genes differentially expressed in CTE OLs versus controls revealed 316 differentially expressed genes between conditions, including: alpha-crystallin B chain (*CRYAB*, *P* = 2.85 × 10^–95^), ferritin heavy chain *(FTH1*, *P* = 3.54 × 10^–85^), and ferritin light chain (*FTL*, *P* = 4.26 × 10^–31^) (Fig. 2f, SI 3).

### Iron accumulation and markers of cellular stress response characterize a specific OL subgroup in CTE

**Fig. 3:**
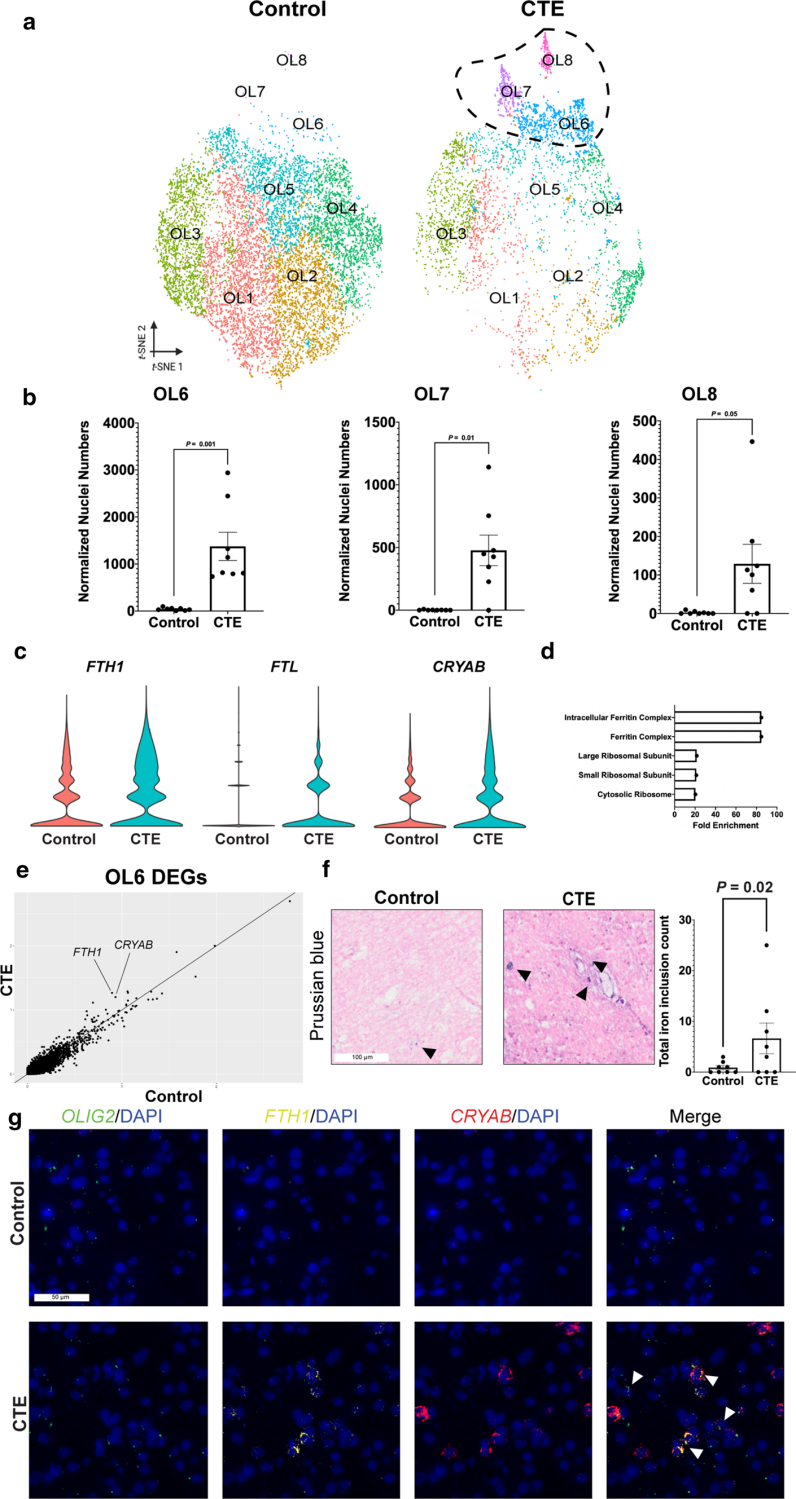
Iron accumulation and markers of cell stress characterized OLs in CTE **a** tSNE projection of mature OL lineage nuclei subset from primary tSNE from Fig. 1b with CTE-specific subpopulations circled. **b** Scatter plot with bar for normalized nuclei counts for CTE-specific OL subpopulations, each dot represents one subject (*n* = 8 per condition; OL6: *P* = 0.001, *U* = 0.0; OL7: *P* = 0.01, *U* = 5.5; OL8: *P* = 0.05, *U* = 11.0; FDR-corrected two-tailed Mann–Whitney *U* test). **c** Top 5 enriched gene ontology terms for OLs in CTE ranked by fold enrichment. **d** Violin plots of log-normalized counts for top expressed genes in CTE-specific OLs (OL6, OL7, and OL8) versus OL1-OL5 in both controls and CTE. **e** Scatter plot for average gene expression in OL6. Control gene expression across the *x*-axis, CTE gene expression across the *y*-axis. *FTH1* and *CRYAB* are labeled as highly differentially expressed genes between conditions. **f** Representative images of Prussian blue staining for control and CTE tissue. Black arrows label iron inclusions. Scale bar (left, white): 100 µm. Quantification (right) for total iron inclusion count, each dot represents a subject (*n* = 8 per condition, *P* = 0.04, one-tailed Student’s t test, *t*(14) = 1.878). g. Representative multiplexed smFISH images for *OLIG2* (green), *FTH1* (yellow), *CRYAB* (red), and DAPI (blue). *OLIG2* and DAPI (left), *FTH1* and DAPI (middle-left), *CRYAB* and DAPI (middle-right), and merge of all probes (right). White arrows label nuclei positive for *OLIG2*, *FTH1*, *CRYAB*, and DAPI. Scale bar (top-left, white): 50 µm. All data were presented in Mean ± SEM

Having observed aberrant OL subpopulations in CTE, we examined whether these differences were disease-specific. We first considered the contrast of fewer overall OLs to the apparent increases in OL cell numbers in certain subpopulation clusters in CTE versus controls (Fig. 3a). To determine whether these OL subpopulations consistently harbored more cells in CTE samples, we compared normalized nuclei counts between CTE and control (Fig. 3b, SI 5, Methods). All three CTE-specific OL subpopulations (OL6, OL7, and OL8) were significantly elevated in CTE compared to control (OL6: *P* = 0.001, OL7: *P* = 0.01, OL8: *P* = 0.05, two-tailed non-parametric Mann–Whitney *U* test) (Fig. 3b).

To determine whether differentially expressed genes in CTE OLs were associated with dysregulated gene modules, we performed gene ontology (GO) analysis [42] using the identified differentially expressed genes in OLs in CTE versus controls. GO analysis revealed a significant increase in gene modules related to iron, iron binding, mRNA translation, and ribosomal processes (Fig. 3c, SI 4).

With the emergence of CTE-specific subpopulations in contrast with an overall reduction of OLs in CTE, we wondered whether the CTE-specific subpopulations may be driving the observed increase in gene transcripts associated with iron accumulation and cellular stress response. We compared the gene expression profiles of CTE-specific OL subclusters (OL6, OL7, and OL8) to all other OL subclusters in both CTE and control and identified three differentially expressed genes associated with aberrant iron accumulation (*FTH1, P* = 1.03 × 10^–73^) and cellular stress response (*CRYAB, P* = 2.31 × 10^–88^; *HSP90AA1, P* = 1.27 × 10^–18^) (Fig. 3d, SI 3) [10, 26, 44, 56]. We then visualized the gene expression profiles of each OL subpopulation individually (Appendix Fig. 9, SI 2). Interestingly, the most compelling upregulation of iron accumulation genes and cellular stress response genes occurred in the OL6 subpopulation, a subpopulation that is significantly elevated in CTE tissue as compared to control (Fig. 3e). The OL6 subpopulation, when compared to every other OL subcluster, expressed significantly more transcripts for *FTH1* (*P* = 6.15 × 10^–66^), *CRYAB* (*P* = 2.79 × 10^–120^) and *HSP90AA1* (*P* = 2.37 × 10^–31^).

To independently validate the presence of excess iron accumulation in CTE, we analyzed the tissue histochemically for iron aggregates (hemosiderin) [26, 46] (Fig. 3f, left, Appendix Fig. 10a, b). We observed an increase in the total number of iron aggregates in CTE white matter compared to control (*P* = 0.04, one-tailed parametric Student’s *t* test) (Fig. 3f, right). By neuropathological evaluation of both CTE and controls, we observed a greater number of white matter perivascular macrophages in CTE samples (Table 1), supporting the presence of aberrant iron accumulation in CTE. Finally, to validate the presence of OLs with abundant *FTH1* and *CRYAB* transcripts, we performed multiplexed smFISH on all control and CTE subjects (Fig. 3g, Appendix Fig. 6c). Due to an overall reduced number of OLs in CTE tissue compared to control tissue (Fig. 2), we identified only sparse groups of *OLIG2* positive nuclei in CTE (Fig. 2d). Remaining OLs in CTE tissue often demonstrated positivity for *FTH1* and *CRYAB*, consistent the OL6 subpopulation (Fig. 3g, Appendix Fig. 10c).

### Identification of neuroinflammatory astrocytes in CTE white matter

**Fig. 4:**
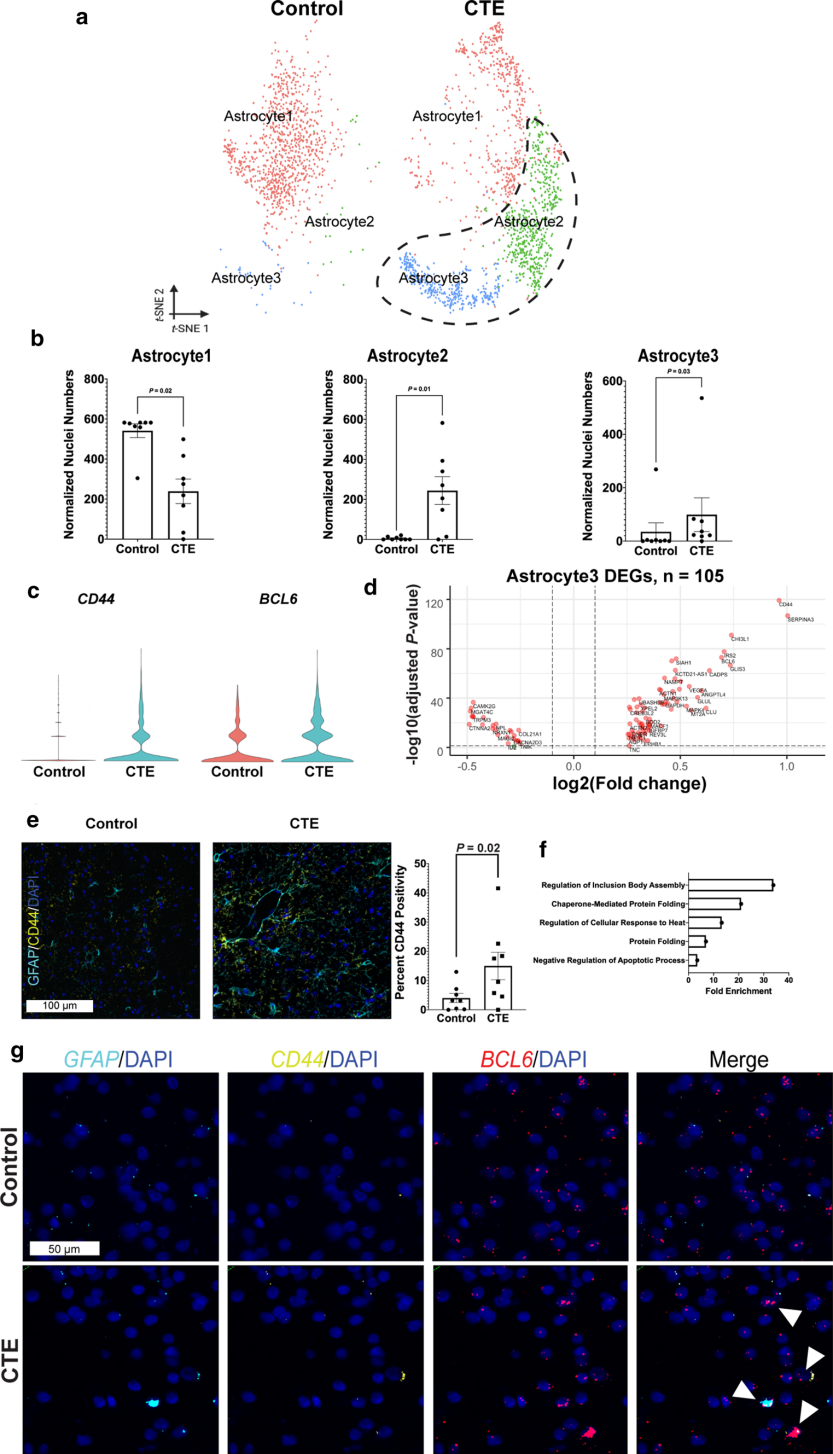
Neuroinflammatory astrocytes in CTE white matter **a** tSNE projection of astrocyte nuclei subset from primary tSNE (Fig. 1b). **b** Scatter plot with bar for normalized nuclei counts for astrocyte subpopulations, each dot represents one subject (*n* = 8 per condition; Astrocyte1: *P* = 0.02, *U* = 2.0*;* Astrocyte2: *P* = 0.01, *U* = 7.5*;* Astrocyte3: *P* = 0.03, *U* = 12.0; FDR-corrected two-tailed Mann–Whitney *U* test). **c** Violin plots of log-normalized counts for top expressed genes in CTE-specific astrocytes. **d** Volcano plot for differentially expressed Astrocyte3 genes between conditions (Bonferroni correction for multiple comparisons). Vertical dashed lines indicate log2(Fold change) cut-off of 0.01. Horizontal dashed lines indicate −log10(adjusted *P* value) cut-off of 0.05. Red dots are CTE-associated differentially expressed Astrocyte3 genes that pass both cutoffs. *n* = 105 differentially expressed genes. **e** Representative IF images for GFAP (teal), CD44 (yellow), and DAPI (blue) in control and CTE. Scale bar (left, white): 100 µm. Quantification (right) for percent of total area positive for CD44 IF for each subject (*P* = 0.02*,* one-tailed Student’s t test). **f** Top 5 enriched gene ontology terms for astrocytes in CTE ranked by fold enrichment. All data were presented in Mean ± SEM. **g** Representative multiplexed smFISH images for *GFAP* (teal), *CD44* (yellow), *BCL6* (red), and DAPI (blue). *GFAP* and DAPI (left), *CD44* and DAPI (middle-left), *BCL6* and DAPI (middle-right), and merge of all probes (right). White arrows label nuclei positive for *GFAP*, *CD44*, *BCL6*, and DAPI. Scale bar (top-left, white): 50 µm

In our primary tSNE projection, we observed a potential shift in the heterogeneity of astrocytes in CTE compared to control (Fig. 1b, middle). To further explore this apparent shift in astrocyte heterogeneity, we re-projected each astrocyte nucleus data point as a subset tSNE projection from the primary tSNE projection as described above for OLs (Fig. 4a) and observed two CTE-specific astrocyte subpopulations, designated Astrocyte2 and Astrocyte3 (Fig. 4a). We found a significant decrease in the number of normalized Astrocyte1 nuclei and a significant increase in the number of normalized Astrocyte2 and Astrocyte3 nuclei in CTE as compared to controls (Astrocyte1: *P* = 0.02, Astrocyte2: *P* = 0.01, Astrocyte3: *P* = 0.03, two-tailed non-parametric Mann–Whitney *U* test, FDR-corrected) (Fig. 4b, SI 5, Methods). To reveal the transcripts driving the emergence of each subpopulation, we performed cell-type marker analysis (SI 2). The Astrocyte1 group expressed many genes associated with normal functioning astrocytes (e.g. *GFAP,* catenin delta-2 (*CTNND2*)*,* aquaporin-4 (*AQP4*)), while the Astrocyte2 group expressed transcripts genes associated with dysfunctional metabolism (e.g. pyruvate dehydrogenase kinase 4 (*PDK4*) [28]), and the Astrocyte3 group showed enrichment for *CD44*, *BCL6*, and *SERPINA3* transcripts [33, 47] (SI 2). Differential gene expression analysis between CTE and control astrocytes identified an upregulation of transcripts associated with neuroinflammation and aging: cluster of differentiation 44 (*CD44*, *P* = 9.32 × 10^–8^), B-cell lymphoma 6 (*BCL6, P* = 7.14 × 10^–15^), and alpha 1-antichymoptrypsin (*SERPINA3, P* = 5.68 × 10^–35^) (Fig. 4c, SI 3) [32, 33], and additional gene expression analysis for the Astrocyte3 group transcript profile confirmed increases in mRNA transcripts related to neuroinflammation (*SERPINA3, P* = 2.21 × 10^–163^; *CD44, P* = 5.38 × 10^–132^; *BCL6, P* = 2.43 × 10^–77^) (Fig. 4d, SI 2).

By immunofluorescence, we observed a significant increase in total area of CD44 immunopositivity in CTE compared to control (*P* = 0.02, one-tailed parametric Student’s t test) (Fig. 4e, right). Gene ontology (GO) analysis [42] using the identified differentially expressed genes in astrocytes between conditions found a significant increase in gene modules related to processes such as protein folding, heat response, and regulation of apoptosis (Fig. 4f, SI 4) [33]. Further, using multiplexed smFISH, we detected *GFAP*, *CD44*, and *BCL6* positive nuclei consistent with the gene expression profile of Astrocyte 3 and consistent with the gene modules identified by GO analysis (Fig. 4g, Appendix Fig. 12b).

Total astrocyte cell numbers between CTE and control were indistinguishable by snRNA-seq analysis (*P* = 0.29, two-tailed non-parametric Mann–Whitney *U* test) (Appendix Fig. 11b) [40] and confirmed by smFISH (*P* = 0.50, one-tailed non-parametric Mann Whitney U-test) (Appendix Fig. 11d). In addition, the overall area of GFAP immunopositivity was indistinguishable between CTE and control (*P* = 0.44, one-tailed parametric Student’s t test) (Appendix Fig. 11c, Appendix Fig. 12a), indicating no grossly detectable morphological change between astrocytes derived from CTE subjects as compared to astrocytes derived from control subjects (Appendix Fig. 7b, *P* = 0.29, two-tailed non-parametric Mann–Whitney *U* test; and, Appendix Fig. 11d, *P* = 0.50, one-tailed non-parametric Mann–Whitney *U* test).

### Differential gene expression in OPCs and microglia

Further findings related to iron trafficking and iron oversaturation were present within glia in the dataset. In microglia, we detected fewer transcripts associated with transferrin (*TF*; *P* = 5.41 × 10^–19^) and fatty acid 2-hydroxylase (*FA2H*, *P* = 6.90 × 10^–6^) (SI 3) in CTE compared to control [31]. We observed fewer transcripts associated with *TF* in CTE astrocytes compared to control astrocytes, as well (*P* = 0.008) (SI 3). In OPCs, we detected a greater abundance of transcripts encoding human leukocyte antigen A (HLA-A; *P* = 0.024) in CTE compared to controls (SI 3).

## Discussion

We performed a transcriptomic analysis of DLF white matter from 16 male subjects, eight with neuropathologically-verified CTE (stage II and stage III) and eight age- and PMI-matched neurological and neuropathological controls and report the analyses of 24,735 cell nuclei. We found fewer oligodendroglia in CTE compared to controls and differences in oligodendroglial and astrocyte subpopulations. The transcriptomically unique profiles distinguishing OL subtypes and/or OL cell states in CTE point to an enrichment of phenotypes involving cell stress response and iron accumulation (*CRYAB* [44] and *FTH1* transcripts*,* respectively [26, 56]). The data reported here and validated across multiple dimensions–snRNA-seq, smFISH, and IHC/IF–suggest a role for OLs in the pathogenesis of CTE. We identified a reduced number of OLs in CTE tissue compared to control, as well as the emergence of three CTE-specific OL subpopulations. By differential gene expression analysis between OLs in CTE and OLs in control, we observed a significant increase in genes associated with excess iron accumulation and cellular stress response in CTE tissue. We also found iron related transcriptional changes in several glial cell types suggesting that alterations in brain iron, iron transport or iron processing might play a role in CTE neurodegeneration.

Previous snRNA-seq analyses in both TBI and another neurodegenerative tauopathy, Alzheimer’s disease (AD), have also identified OL abnormalities. Mice have reduced numbers of hippocampal OLs and an upregulation of *Tf* in all OLs as an acute response to TBI [4, 45]. Upregulation of *Tf* in murine OLs is likely part of cellular maturation and is a compensatory measure to replace acutely depleted OLs [50]. Myelination-related transcriptional changes have been identified in previous transcriptomic analyses of cerebral cortex in AD. Regulators of myelination, like leucine rich repeat and Ig domain containing 1 (*LINGO1*), have widespread abnormalities in expression across neuronal and glial cell types in AD [36]. Cortical OLs in AD express fewer transcripts associated with myelination, axonal guidance, and maturation of myelin (semaphorin-3B (*SEMA3B*), microRNA 219a-2 (*MIR219A2*), and stathmin 4 (*STMN4*)) [57].

With respect to astrocytes, our transcriptomic analysis of DLF white matter in CTE suggests enrichment of cell subtypes and/or states associated with neuroinflammation and dysfunctional metabolism, without a change in overall astrocyte number. These findings add to the growing body of evidence indicating astrocytic alterations in CTE white matter, including previous studies showing diffuse astrocytosis and astrocytic degeneration in moderate-severe CTE [25] and interface astrogliosis [49].

All the CTE cases in our study experienced decades of exposure to RHI in professional or collegiate football or boxing and mixed martial arts, whereas, these data were unavailable for the controls. It is possible that the differences in oligodendroglia number and oligodendroglial and astrocyte subpopulations found in the CTE cases were related to prolonged RHI exposure and not CTE. Future studies are planned to determine whether these changes occur after RHI alone and whether they increase in severity with increased severity of p-tau pathology and progression of CTE. Nonetheless, these snRNA-seq findings, in combination with our analyses and the evidence in the literature, implicate OLs in CTE, AD and in response to TBI and RHI.

The cell number distribution presented here aligns with the only previous study examining human white matter using snRNA-seq [27], and we did not observe abundant transcriptomic or cell-type differences associated with neurons, microglia, or endothelial cells, likely due to under sampling of those cell nuclei. OLs are abundant in human white matter tissue and the present dataset contains over seven-fold more OLs than astrocytes, the other cell type with notable transcript level differences between CTE and control samples. Given the difficulty in capturing microglia and endothelial cells using snRNA-seq [54], additional transcriptomic investigations of these cells in the white matter of CTE are warranted, especially in light of previous studies showing increases in neuroinflammatory microglia in DLF cortex in CTE [11–13]. Future studies isolating microglia, neurons, and endothelial cells for in depth transcript profiling are also warranted [3, 51]. Furthermore, additional studies specifically isolating astrocyte nuclei for sequencing will enhance the findings presented here [5].

This study has several limitations, including the small sample size and the lack of data regarding RHI exposure in the controls. Additional studies are needed to determine whether similar alterations occur after RHI in the absence of CTE and whether years of exposure correlates with the severity of the change. Larger studies using cases with a wide range of age at death and disease severity would also help define the temporal evolution of the cell number and transcriptomic alterations in the dataset. Furthermore, the small sample size precludes determinations regarding the contribution of oligodendroglial and astrocytic alterations to clinical symptomatology and whether changes in white matter integrity observed microscopically directly relate to the RNA-seq findings. Nonetheless, the presented dataset serves as a mineable resource for understanding the molecular and cellular dysregulation associated with CTE and RHI. Integration with additional datasets will enable expansion of CTE transcriptomic profiles to further characterize potential mechanisms of CTE pathogenesis and provide a catalyst for the future development of biomarkers to aid in the diagnosis of CTE during life as well as potential therapeutics.

### Supplementary Information

Below is the link to the electronic supplementary material.

Supplementary file1 (XLSX 17 KB)

Supplementary file2 (XLSX 9 KB)

Supplementary file3 (XLSX 1109 KB)

Supplementary file4 (XLSX 208 KB)

Supplementary file5 (XLSX 648 KB)

Supplementary file6 (XLSX 29 KB)

Supplementary file7 (XLSX 15831 KB)

Supplementary file8 (XLSX 15198 KB)

## Figures and Tables

**Fig. 5:**
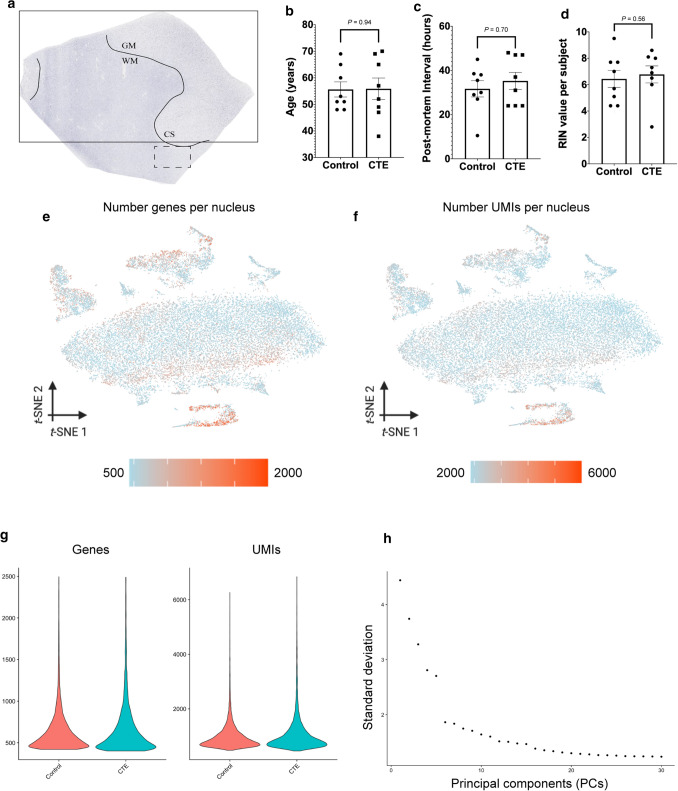
Tissue collection and quality control **a** Image of dorsolateral frontal white matter and depth of a cortical sulcus representing the region of tissue used for this project. Dashed rectangle represents a region taken for snRNA-seq analysis (only white matter at the depth of a cortical sulcus), solid rectangle represents a region taken for all in situ validation (gray and white matter adjacent to the region taken for snRNA-seq). *GM* gray matter, *WM* white matter, *CS* cortical sulcus. **b** Scatter plot with bar for each subject by age (*n* = 8 per condition, median of CTE = 54.5 years, median of control = 52.0 years, *P* = 0.94, two-tailed Mann–Whitney *U* test, *U* = 31.0). **c** Scatter plot with bar for each subject by post-mortem interval (PMI) (*n* = 8 per condition, median of CTE = 34.0 h, median of control = 32.75 h, *P* = 0.70, two-tailed Mann–Whitney *U* test, *U* = 25.0). **d** Scatter plot with bar for each subject by RNA integrity number (RIN) value (*n* = 8 per condition, median of CTE = 7.1, median of control = 6.4, *P* = 0.56, two-tailed Mann–Whitney *U* test, *U* = 26.0). **e** tSNE projection colored by number of genes present in each nucleus. **f** tSNE projection colored by number of unique molecular identifiers (UMIs) present in each nucleus. **g** (left) Violin plots for the number of genes expressed in each nucleus for control and CTE subjects. (Right) Violin plots for the number of UMIs present in each nucleus for control and CTE subjects. **h** Elbow plot for the amount of variance produced by each principal component. Principal components (PCs) from 1 to 30 on the *x*-axis and standard deviation on the *y*-axis. All data were presented in Mean ± SEM

**Fig. 6:**
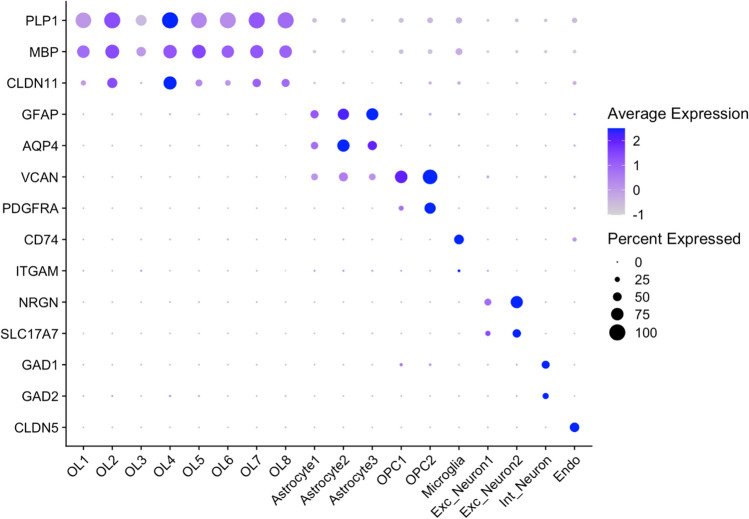
Cell-type-specific marker gene expression Dotplot with cell types on the *x*-axis and marker genes on the *y*-axis. Size of dot relates to the percent of nuclei in a given cluster that express the corresponding marker gene. Color of dot relates to the average marker gene expression for the nuclei expressing the marker gene in a given cluster

**Fig. 7:**
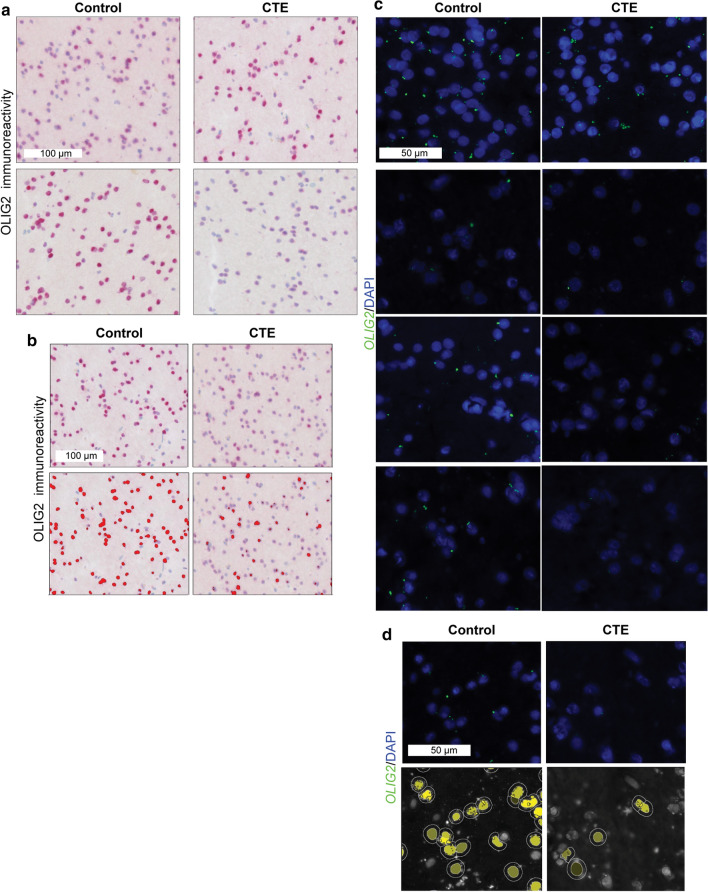
Validation of identification of fewer oligodendrocytes in CTE white matter compared to control **a** Representative immunohistochemistry images for control and CTE stained with anti-OLIG2 for OL lineage cells. Scale bar (top-left, white): 100 µm. **b** Immunohistochemistry images from main text (top) and the same images with masks (bottom) to demonstrate how positive cells are identified. Scale bar (top-left, white): 100 µm. **c** Representative smFISH images for *OLIG2* (green) and DAPI (blue). Scale bar (top-left, white): 50 µm. **d** smFISH images from main text (top) and the same images with masks (bottom) to demonstrate how nuclei and positivity are identified. Scale bar (top-left, white): 50 µm

**Fig. 8:**
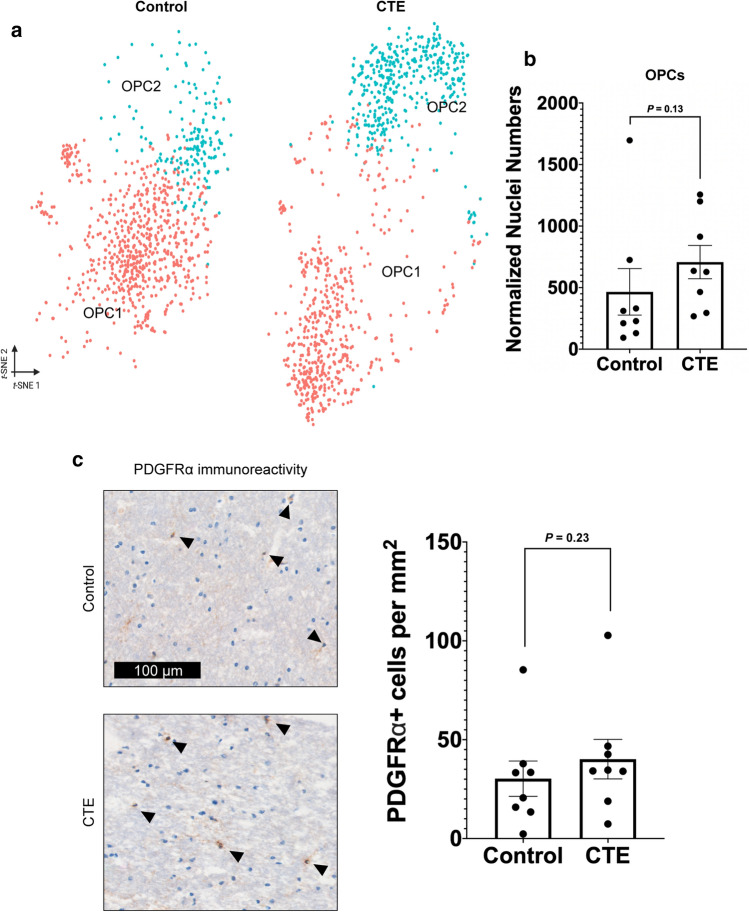
OPC cell numbers do not change between conditions **a** tSNE projection of OPC nuclei subset from primary tSNE from Fig. 1b. **b** Scatter plot with bar for normalized nuclei counts for all OPC nuclei, each dot represents the total normalized number of OPC nuclei in one subject (*n* = 8 per condition, *P* = 0.13, FDR-corrected two-tailed Mann–Whitney *U* test, *U* = 17.0). **c** Representative immunohistochemistry images for control and CTE stained with anti-PDGFRα for OPCs. Scale bar (top, black): 100 µm. Quantification (right) for the number of PDGFRα-positive cells per mm^2^ over the entire white matter sample for each subject (*n* = 8 per condition, *P* = 0.24, one-tailed Student’s t test, *t*(14) = 0.7363). Black arrows label cells positive for PDGFRα. All data were presented in Mean ± SEM

**Fig. 9:**
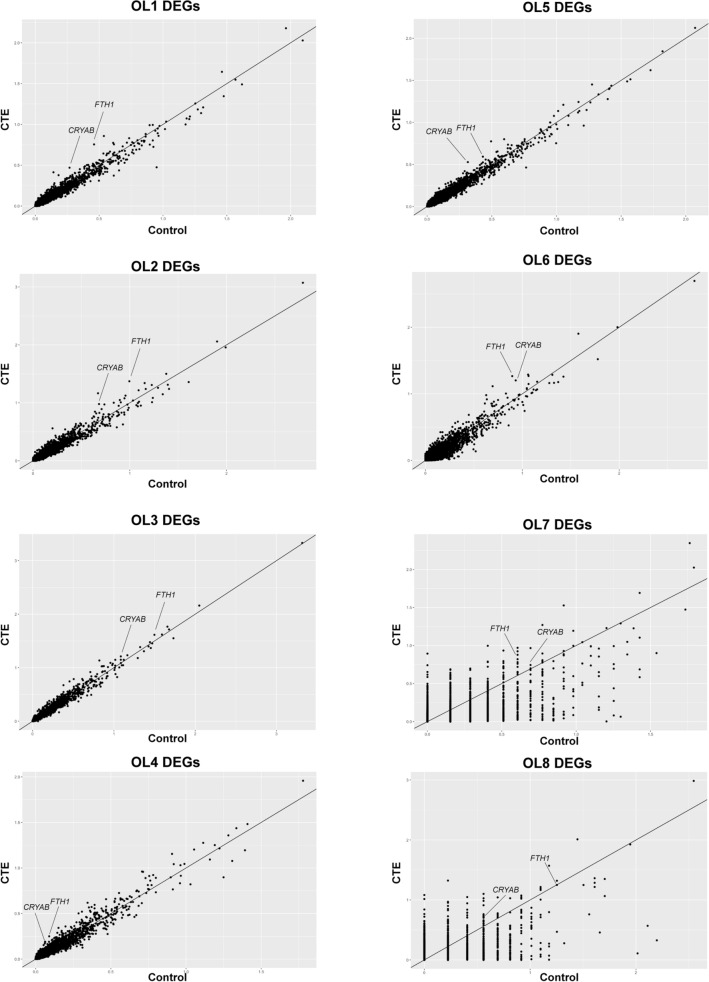
Average gene expression in CTE and control conditions across OL subtypes Scatter plot for average gene expression in all 8 OL subtypes. Control gene expression across the *x*-axis, CTE gene expression across the *y*-axis. *FTH1* and *CRYAB* are labeled

**Fig. 10:**
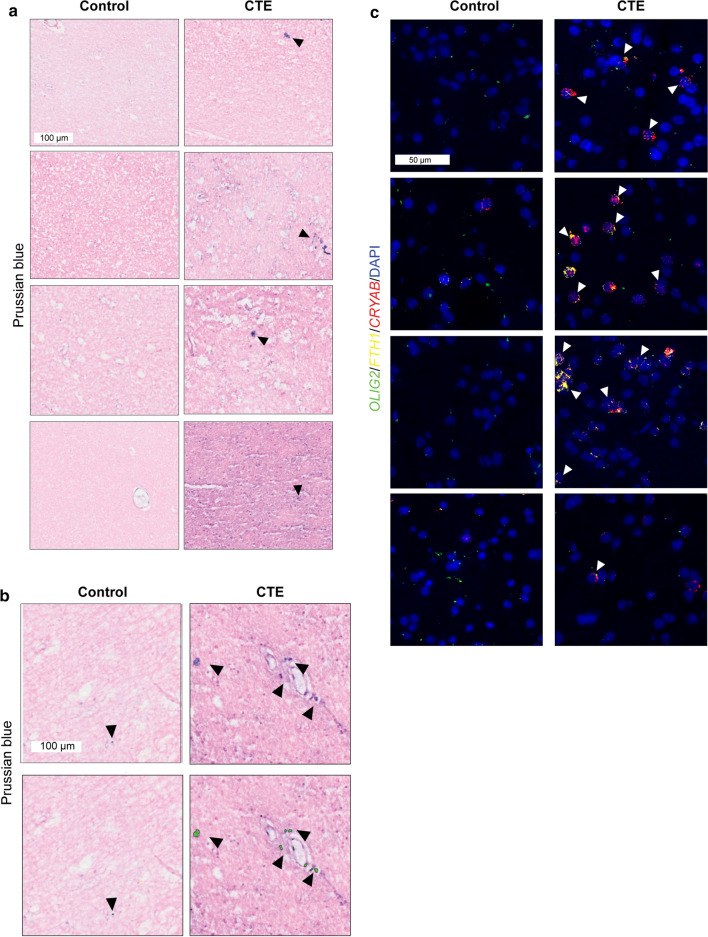
Validation of iron accumulation in CTE-specific OLs **a** Representative images of Prussian blue staining for control and CTE. Black arrows label iron inclusions. Scale bar (top-left, white): 100 µm. **b** Prussian blue images from main text (top) and the same images with masks to demonstrate how iron inclusions are identified (bottom). Scale bar (top-left, white): 100 µm. **c** Representative multiplexed smFISH images for *OLIG2* (green), *FTH1* (yellow), *CRYAB* (red), and DAPI (blue). White arrows label nuclei positive for *OLIG2*, *FTH1*, *CRYAB*, and DAPI. Scale bar (top-left, white): 50 µm

**Fig. 11:**
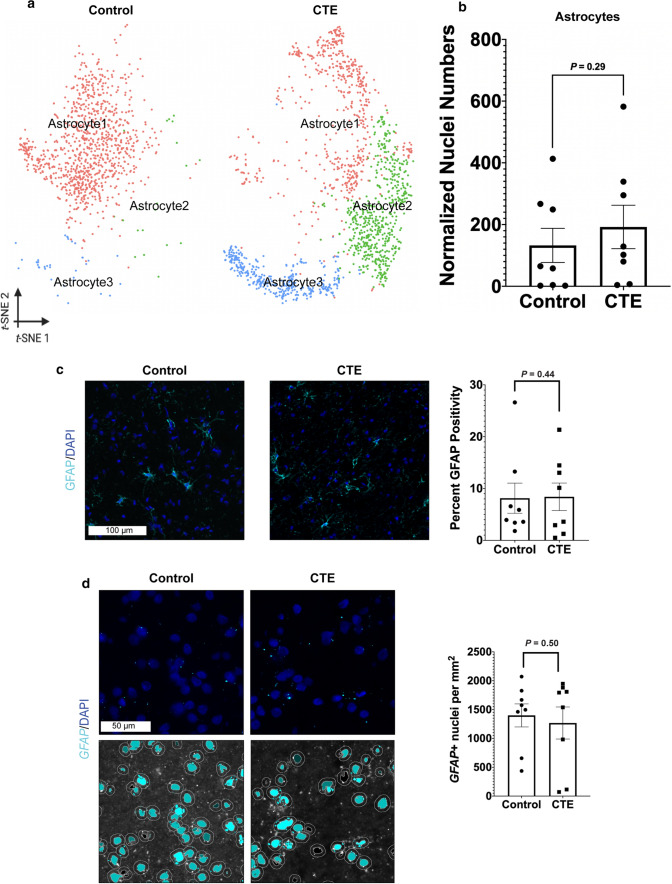
Astrocyte cell numbers do not change between conditions **a** tSNE projection of astrocyte nuclei subset from primary tSNE from Fig. 1b. **b** Scatter plot with a bar for normalized nuclei counts for all astrocytes, each dot represents the total normalized number of astrocyte nuclei in one subject (*n* = 8 per condition, *P* = 0.29, FDR-corrected two-tailed Mann–Whitney *U* test, *U* = 0.29). **c** Representative IF images for GFAP (teal) and DAPI (blue) in control and CTE. Scale bar (left): 100 µm. Quantification (right) for percent of total area positive for GFAP IF for each subject (*P* = 0.44*,* one-tailed Student’s t test). **d** (top) Representative smFISH images for *GFAP* (teal) and DAPI (blue). (Bottom) Same images as top but with masks to demonstrate how nuclei and positivity are identified. Scale bar (top-left, white): 50 µm. Quantification (right) for number of *GFAP*-positive nuclei for each subject (*n* = 8 per condition, median of CTE = 1667, median of control = 1536, *P* = 0.50*,* one-tailed Mann Whitney *U* test, *U* = 32.0). All data were presented in Mean ± SEM

**Fig. 12:**
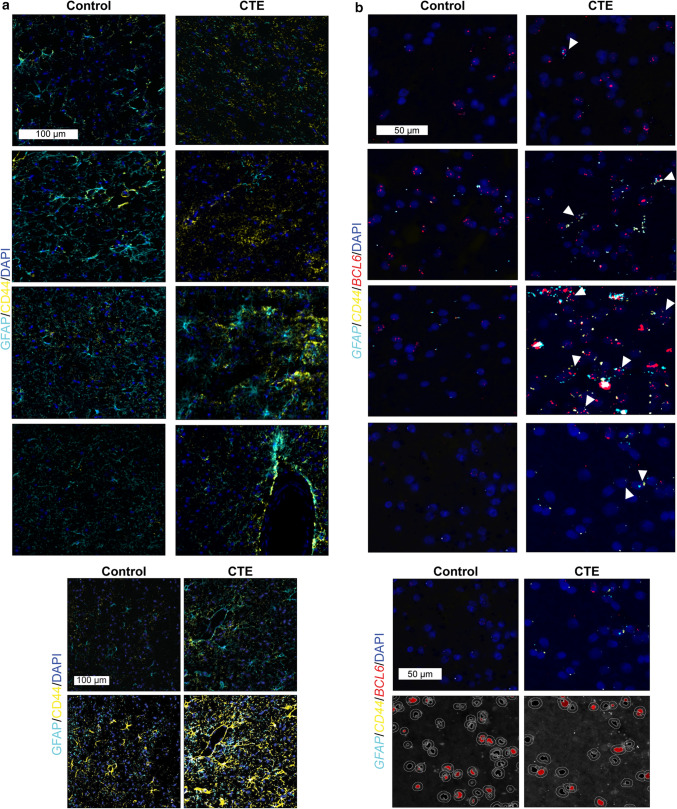
Validation of neuroinflammatory astrocytes in CTE white matter **a** Representative IF images for GFAP (teal), CD44 (yellow), and DAPI (blue) in control and CTE. Scale bar (top-left, white): 100 µm. **b** IF images from main text (top) and the same images with masks (bottom) to demonstrate how GFAP and CD44 positivity are identified. Scale bar (top-left, white): 100 µm. **c** Representative multiplexed smFISH images for *GFAP* (teal), *CD44* (yellow), *BCL6* (red), and DAPI (blue) in control and CTE. White arrows label nuclei positive for *GFAP*, *CD44*, *BCL6*, and DAPI. Scale bar (top-left, white): 50 µm. **d** Multiplexed smFISH images for *GFAP* (teal), *CD44* (yellow), *BCL6* (red), and DAPI (blue) in control and CTE (top) and the same images with masks (bottom) to demonstrate how nuclei and *BCL6* positivity are identified. Scale bar (top-left, white): 50 µm

## Appendix

See Figs. 5, 6, 7, 8, 9, 10, 11, 12.
